# Time course of auditory streaming: do CI users differ from normal-hearing listeners?

**DOI:** 10.3389/fpsyg.2014.00775

**Published:** 2014-07-21

**Authors:** Martin Böckmann-Barthel, Susann Deike, André Brechmann, Michael Ziese, Jesko L. Verhey

**Affiliations:** ^1^Department of Experimental Audiology, Faculty of Medicine, Otto von Guericke University MagdeburgMagdeburg, Germany; ^2^Special Lab Non-invasive Brain Imaging, Leibniz Institute for NeurobiologyMagdeburg, Germany

**Keywords:** cochlear implant, auditory streaming, build-up, perception, psychoacoustics

## Abstract

In a complex acoustical environment, the auditory system decides which stimulus components originate from the same source by forming auditory streams, where temporally non-overlapping stimulus portions are considered to originate from one source if their stimulus characteristics are similar. The mechanisms underlying streaming are commonly studied by alternating sequences of A and B signals which are often tones with different frequencies. For similar frequencies, they are grouped into one stream. Otherwise, they are considered to belong to different streams. The present study investigates streaming in cochlear implant (CI) users, where hearing is restored by electrical stimulation of the auditory nerve. CI users listened to 30-s long sequences of alternating A and B harmonic complexes at four different fundamental frequency separations, ranging from 2 to 14 semitones. They had to indicate as promptly as possible after sequence onset, if they perceived one stream or two streams and, in addition, any changes of the percept throughout the rest of the sequence. The conventional view is that the initial percept is always that of a single stream which may after some time change to a percept of two streams. This general build-up hypothesis has recently been challenged on the basis of a new analysis of data of normal-hearing listeners. Using the same experimental paradigm and analysis, the present study found that the results of CI users agree with those of the normal-hearing listeners: (i) the probability of the first decision to be a one-stream percept decreased and that of a two-stream percept increased as Δf increased, and (ii) a build-up was only found for 6 semitones. Only the time elapsed before the listeners made their first decision of the percept was prolonged as compared to normal-hearing listeners. The similarity in the data of the CI user and the normal-hearing listeners indicates that the quality of stream formation is similar in these groups of listeners.

## INTRODUCTION

Making sense of the complex auditory environment is of eminent importance in life. For hearing-impaired listeners and for those with their hearing restored by a cochlear implant (CI), a task such as the focusing on a conversational partner in a noisy environment often poses serious problems. Since the pioneering work of Miller and Heise (1950), Bregman and Campbell (1971), and van Noorden (1975), laboratory experiments have used the auditory streaming paradigm to understand the processes that help to disentangle several sound sources. In this paradigm, tones from two sets A and B differing in a specific sound feature are presented in rapid alternation, and listeners are asked if they hear one stream or two co-existing streams. Since fundamental frequency is the sound feature most often used to study the formation of streams, in the following, stream formation is discussed in relation to this feature only. At sufficiently high presentation rates, a two-stream percept is basically possible if the frequency separation Δ*f* of the two sets exceeds a minimum value, the so-called fission boundary (cf. van Noorden, 1975). Whereas for large frequency separations, the segregated percept predominates, at intermediate frequency separations the perceptual organization is ambiguous, that is, both integration into one stream and segregation into two streams are possible (see Bregman, 1990, for a review).

The type of percept may change over the duration of a sequence. It has been generally assumed that “ABAB” sequences of alternating A and B sounds are initially heard as one stream, and that only after some time they are perceived as two separate streams which the listener can follow separately (see, e.g., Bregman, 1978; Anstis and Saida, 1985; Carlyon et al., 2001; Micheyl et al., 2005; Pressnitzer et al., 2008). Recently, Deike et al. (2012) used harmonic tone complexes in an ABAB sequence to study segregation in normal-hearing listeners. The average frequency separation between A and B tones ranged from 2 to 14 semitones. The rate was set to 6 tones per second. Listeners were asked to indicate as soon as possible their current percept and any change of it over the duration of the presented sequence. The probability of the one-stream and the two-stream percepts was tracked independently, thus providing the current probability of segregation unbiased toward any type of initial percept. They observed an increase in the two-stream probability only for the most ambiguous stimulus condition with a frequency separation of 6 semitones between the A and B harmonic complexes. At larger frequency separations, the first percept was that of two-stream, in contrast to the general assumption of an initial one-stream percept. The probability of a two-stream percept was above 80% at the beginning of the sequence and hardly changed over the course of the sequence. This finding is in agreement with a recent study by Denham et al. (2013), who also found an initial two-stream percept at large frequency separations and high presentation rates. Based on their results in normal-hearing listeners, Deike et al. (2012) argued that a build-up of segregation is not generic and requires ambiguity of the sound sequences to occur.

Only few studies investigated stream segregation in CI users (Chatterjee et al., 2006; Hong and Turner, 2006; Cooper and Roberts, 2007, 2009; Marozeau et al., 2013). CIs can restore, to a certain extent, hearing in patients with a severe to complete cochlear hearing loss by stimulating the auditory nerve electrically. The signal is picked up by an external microphone and is then mapped to a linear array of 12–22 independent electrodes which was inserted into the cochlea. Each of these electrodes covers a certain frequency range.

All previous studies consistently found that effects related to stream segregation are observed in some of the CI users only. These studies either asked their listeners explicitly what they perceive at the end of the sequence (e.g., Chatterjee et al., 2006) or investigated stream segregation with a task where it is advantageous to perceive one stream (Hong and Turner, 2006; Cooper and Roberts, 2009) or two streams (Marozeau et al., 2013). Some of these studies used direct electrical stimulation of the electrodes, thus investigating if a stimulation of separate parts of the auditory nerve may generate stream segregation (Chatterjee et al., 2006; Cooper and Roberts, 2007, 2009). The others studies presented the stimuli through a loudspeaker and the microphone of the CI device (Hong and Turner, 2006; Marozeau et al., 2013).

A limitation of most studies is that they used short sequences with durations of several seconds where it is not clear if the percept is allowed to fully evolve. Only in one experiment, the sequence was 30 s long without changing the stimulus parameters (Cooper and Roberts, 2007). Unfortunately, in their study, listeners were instructed to press a single response button to indicate when the percept changed without defining the type of percept. Thus, their results do not allow to investigate the initial percept and how this was affected by the frequency separation.

Cooper and Roberts (2007, 2009) argued that their stream segregation results as well as those of Hong and Turner (2006) could be accounted for by assuming that listeners counted the number of different tones requiring a simple discrimination of A and B tones. Due to physiological and technical constraints of the CI, frequency discrimination of these patients is in order of several semitones (see for example, Pretorius and Hanekom, 2008). Thus, if stream segregation (with tone stimuli) is solely based on frequency discrimination, their ability to segregate streams should be worse than in normal-hearing listeners. One aim of this study is to investigate if this restriction results in a different stream formation in CI users compared to normal-hearing listeners.

Marozeau et al. (2013) also used long ABAB sequences of harmonic tone complexes but in contrast to Cooper and Roberts (2007) the frequency separation Δ*f* of the A and B sounds gradually increased or decreased. Listeners were instructed to continuously rate the difficulty to follow the melody of the A tone set. When starting at a large average value of one octave, CI users could follow the melody rather easily, and increased the difficulty rating slowly with decreasing Δ*f*. When increasing Δ*f* from completely overlapping A and B tone sets, however, they found it much harder to hear out the melody, even at a Δ*f* of one octave. Because each of the tone sets itself covered a range of 7 semitones, the results were not easily explained by simple discrimination of A and B tones but suggested an explicit segregation. Since the parameters continually changed in the experiment, the development and stability of a segregated percept could not be obtained from these data.

The main aim of the present study is to investigate the time course of the formation of streams in CI users and if this formation is similar in quality to that observed in normal-hearing listeners. The experiment of Deike et al. (2012) was replicated in experienced CI users with a reduced selection of the same stimuli and the identical task. The time course of perception is analyzed in the conventional way under the assumption of a default one-stream percept in comparison to the new analysis method used in Deike et al. (2012) which does not make any assumption of the percept before the first perceptual decision has been made. The proportions of time over which the sounds were perceived as either one-stream or two-streams are investigated as well as the time needed for the first perceptual decision.

## MATERIALS AND METHODS

### LISTENERS

Eight CI users aged 60–83 years (median age: 69 years), four female and four male, participated in the study. CI experience ranged from 8 months to 16 years (median: 2 years, 1 month). Demographic data of the participants are specified in **Table 1**. All participants used only one implant in the experiments. Bilaterally implanted listeners (CI01, CI06, CI08) usually listened through the earlier implanted side. The exception is listener CI08, who used the more recently implanted device due to significantly better performance with this implant than with the contralateral one. One participant with residual hearing (CI07) was equipped with an attenuating ear plug. All participants used their everyday device settings. The study was approved by the Ethics Committee of the Otto von Guericke University of Magdeburg to conform to the declaration of Helsinki. Each participant provided written informed consent to the study and was compensated for his expenses. Two additional CI users (not included in **Table 1**) had to be excluded from the experiment due to a consequent misunderstanding of the task or because recording of the responses failed due to a technical problem of the experimental setup.

**Table 1:** Demographic data of the CI users participating in the experiment.

ID	Sex	Age (years)	CI experience (years; months)	Side	Contralateral hearing	Implant type	Sound processor	Processing strategy	Number of active channels	Lower cut-off frequency (Hz)
CI01	m	65	02;05	L	CI	Sonata	Opus2	FSP	12	100
CI02	f	83	01;08	R	–	Sonata	Opus2	FS4	12	100
CI03	m	65	16;01	R	–	CI40+	Opus2	FSP	11	100
CI04	m	73	02;01	L	–	Concerto	Opus2	FS4	12	100
CI05	f	69	03;02	L	–	CI512	CP810	ACE	19	188
CI06	f	73	01;08	R	CI	Sonata	Opus2	FS4	12	100
CI07	f	69	00;08	R	Residual	Concerto	Opus2	FS4	11	100
CI08	m	60	15;08	R	CI	CI40+	Opus2	FSP	9	100

*All listeners were studied using only one CI device. The side is specified in the respective column labeled “side.” One listener (CI07) was equipped with an earplug on the contralateral side due to residual hearing.*

### APPARATUS, STIMULI, AND PROCEDURE

Whereas in the previous study on streaming in normal-hearing listeners (Deike et al., 2012) headphones were used, here sounds were presented to the CI users in a free-field condition in a sound-attenuated room through a single, frontally located active monitor loudspeaker (Reveal 6D, Tannoy Ltd., Coatbridge, UK). The level was individually adjusted to a comfortable level. Stimulus presentation and response collection was administered using Presentation (Neurobehavioral Systems Inc., San Francisco, CA, USA).

Otherwise, stimulus material and presentation were identical to that of Deike et al. (2012). Tones from a high-frequency set A and a low-frequency set B alternated in an ABAB order at a rate of 6 tones per second. The harmonic tone complexes consisted of the first five partials, including the fundamental frequency, at equal amplitudes. The duration of each tone complex was 25 ms including 3.8-ms cosine-squared onset and offset ramps. To construct ABAB sequences with different frequency separations (Δ*f* ) of A and B tone sets, the fundamental frequencies of both the A and B tones were arranged symmetrically on a semitone scale around 392 Hz. In contrast to Deike et al. (2012), only four conditions instead of seven with average values Δ*f* of 2, 6, 10, and 14 semitones were used. The center values of the fundamental frequencies for the different conditions are given in **Table 2**. In addition, within each condition, individual exemplars of both A and B tones varied in the fundamental frequency in five discrete steps (±2, ±1, and 0 semitones). Forty sequences, each with a certain average Δ*f* and duration of 30 s, were presented in a random order. The tone complexes were generated by MATLAB (The Mathworks Inc., Natick, MA, USA) prior to the experiment. Since the stimulus settings were identical to Deike et al. (2012), their data of normal-hearing listeners were used as a control.

**Table 2:** Median fundamental frequencies fo of the A and B tone complexes in the different Δ*f* conditions.

	fo/Hz	Condition:
		Δ*f*/semitones
**A tones**	587	14
	523	10
	466	6
	415	2

**B tones**	370	2
	330	6
	294	10
	262	14

*In addition, these values are randomly varied in semitone steps over a range of ±2 semitones within the A and B sounds (for details, see text).*

The task of the listeners was to indicate their current percept continuously on a computer mouse. The listeners were asked to press the left button (marked as “1”) as long as a single, see-saw stream of high and low tones (or of more or less the same pitch, termed as “one-stream”) was perceived, and the right button (marked as “2”) as long as a separation into two separate, parallel streams of high tones and low tones (termed as “two-stream”) was perceived. In addition, sketches of an oscillating curve and two parallel curves were shown as graphical analogies of the two percept types prior to the experiment. The listeners were asked to enter a decision as soon as they had one of the two percepts, and to switch to the other button whenever the percept changed to the other type. Prior to the experiment, two sequences with the narrowest separation (Δ*f* = 2 semitones) and two sequences with the widest separation (Δ*f* = 14 semitones) were presented in an alternating manner to familiarize the listeners with the task.

### DATA ANALYSIS

From the recorded instants of the responses, the proportions of time that the sound sequence was perceived as either one-stream or two-stream were calculated for each CI user and each condition. The resulting proportions of time were subjected to repeated-measures analyses of variance (SPSS Version 21, IBM, Armonk, NY, USA), testing for the within-subject factor Δ*f* condition. Where necessary (due to violation of the sphericity assumption), *p* values were corrected according to Greenhouse–Geisser. Since it was expected that the proportion of time the sequences were perceived as two streams increases with increasing Δ*f*, planned contrasts were studied by calculating the polynomic trends.

In addition, for each CI user the median of the time to the first response was calculated across the 10 sequence presentations at each Δ*f* condition. The median was used instead of mean values as the data were skewed toward smaller values, between as well as within listeners. Because the data were thus not normally distributed, a non-parametric Friedman test was conducted. *Post hoc* Wilcoxon tests are reported with Bonferroni correction of multiple testing.

The time course of the probability of each of the two percepts was calculated as described in Deike et al. (2012). Each sequence was divided into 1-s bins. For the probability of a one-stream percept, a value of one was assigned to each bin where the last answer had been a left-mouse button (“1”) press, and a value of zero if it had been a right-mouse button (“2”) press. This probability was averaged across the 10 presentations of a certain Δ*f* value. In the same way, the probability of a two-stream percept was calculated by assigning a value of 1 to each bin where the last answer had been a right-mouse button (“2”) press, and a value of 0 if it had been a left-mouse button (“1”) press. The average across the 10 presentations for a Δ*f* value yields to the time course of the two-stream percept for this frequency separation. In each average time course, the first bins were taken only as long as at least one response of at least two participants had occurred.

## RESULTS

### PROPORTIONS OF PERCEPT TYPES

**FIGURE 1:**
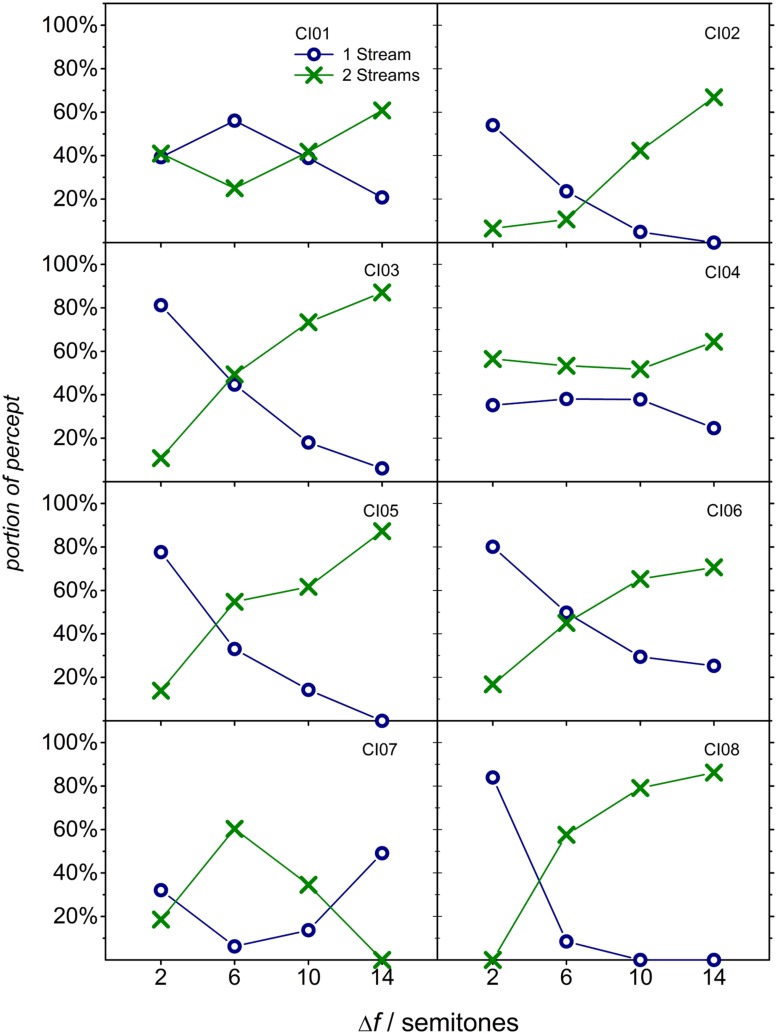
**Individual durations of answering options “two-streams” (green cross marks) and “one-stream” (blue circles), calculated as proportions of the total sequence duration (30 s), are plotted as a function of the (average) Δ*f* for each listener**.

To assess if listeners perceived alternating sequences as one (single) stream or two (segregated) streams at the four different Δ*f* values, the individual proportions of both percepts were calculated over the whole duration of the sequence. In case the listeners showed stream segregation, the proportion of a two-stream percept should increase with Δ*f*, whereas the proportion of a one-stream percept should decrease. The results are displayed in **Figure 1**. In five listeners (CI02, CI03, CI05, CI06, and CI08), an increase of the proportion of the two-stream percept was observed, in agreement with the streaming hypothesis. Three listeners (CI01, CI04, and CI07) had similar proportions of one-stream and two-stream percepts at all Δ*f*, indicating that they did not experience an increase of the two-stream percept in any of the tested conditions.

**FIGURE 2:**
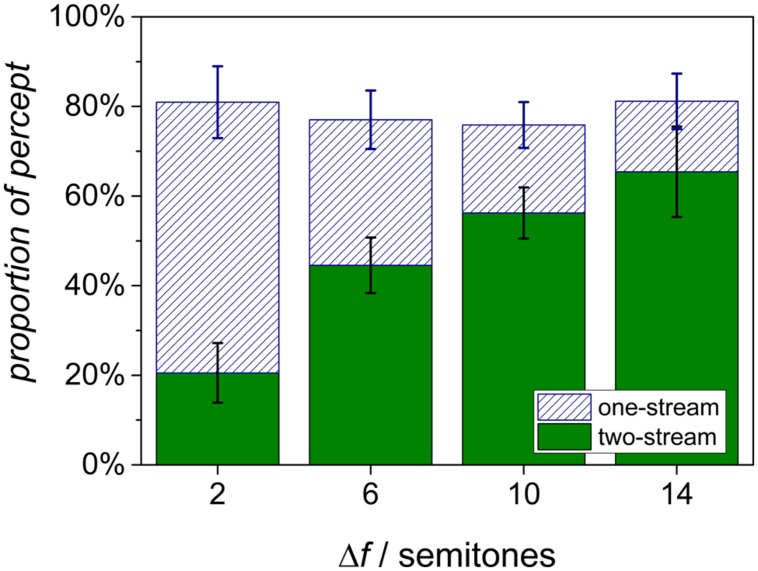
**Grand means of the proportions of the two percepts from eight listeners for different Δ*f* conditions.** Error bars denote the standard error of mean.

The grand means of the data are shown in **Figure 2**. The proportion of the two-stream percept increases with Δ*f*, whereas the proportion of the one-stream percept is reduced. The most ambiguous situation with equivocal proportions of both percepts is found at a Δ*f* of 6 semitones. Note that the summed proportions do not add up to 1. This “missing” proportion represents the time from stimulus onset until the first perceptual decision was reported. The difference to 100% corresponds to the mean reaction time for the first response relative to the total duration.

For the analysis of the mean proportion of the two-stream percept, a repeated-measures analysis of variance was conducted on the factor Δ*f* condition. For the proportion of the two-stream percept, Mauchly’s test indicated the violation of the sphericity assumption. Using Greenhouse–Geisser corrected values (𝜀 = 0.61), this proportion depended with high significance on Δ*f* [*F*(3,21) = 7.39, *p* < 0.01]. Planned contrasts yielded a significantly increasing linear trend of the proportion of the two-stream percept with increasing Δ*f* [*F*(1,7) = 12.30, *p* < 0.01]. The mean proportions of the one-stream percept were analyzed in the same way, using Greenhouse–Geisser correction (𝜀 = 0.58). Here, Δ*f* also had a significant effect [*F*(3,21) = 9.21, *p* < 0.01]. Planned contrasts yielded a significantly decreasing linear trend of the proportion of the one-stream percept with increasing Δ*f* [*F*(1,7) = 9.21, *p* < 0.01].

### FIRST RESPONSE

**FIGURE 3:**
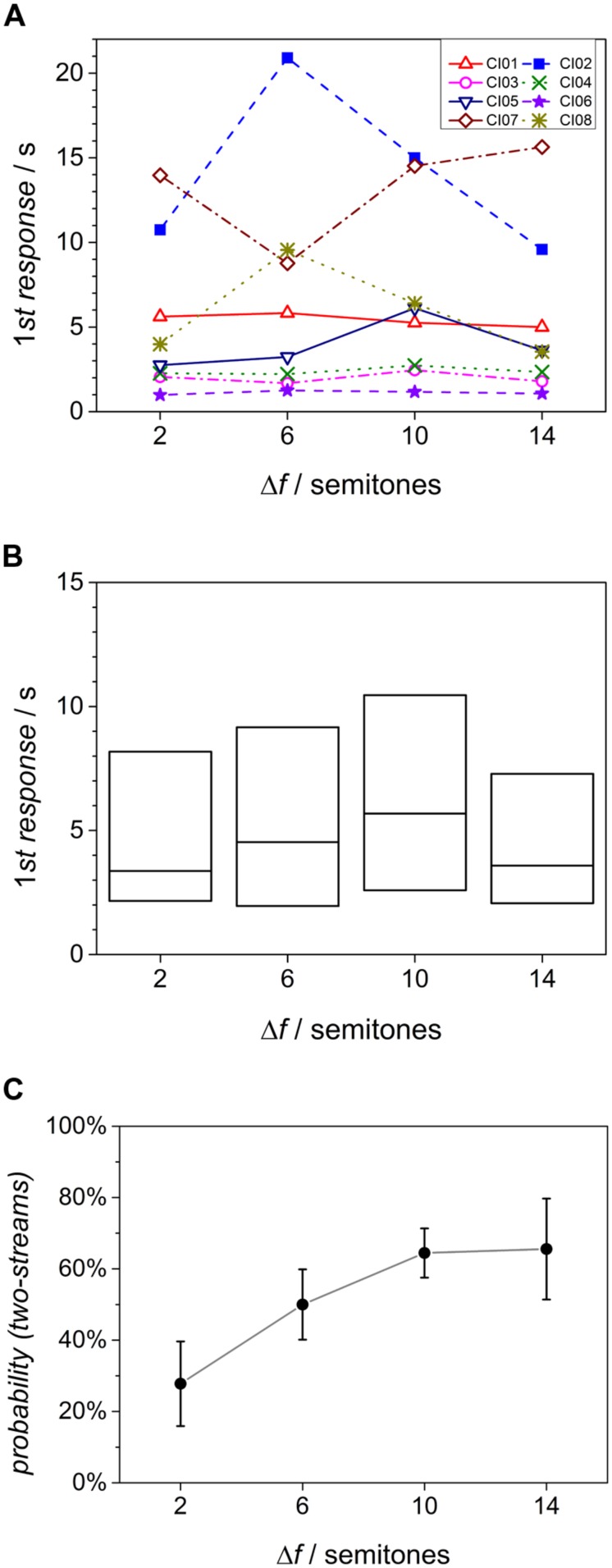
**(A)** Time of the first response; individual values from eight listeners. **(B)** Box plot of the time of first response. Box boundaries mark the lowest and highest quartiles, center lines the median. **(C)** Type of first decision for different Δ*f* conditions.

Each of the listeners provided at least one response within the duration of each single sequence. **Figures 3A,B** displays the time elapsed before the first response as individual data and box plot. Shown are individual values of the eight listeners evaluated before. Only three of the listeners consequently provided the first response within 3 s. Two listeners (CI02 and CI07) reacted very slowly with first button presses after more than 10 s. The effect of the factor Δ*f* on the time of the first response was investigated using a non-parametric, Friedman test for related samples. This yielded no significant effect of Δ*f* [χ^2^(3) = 4.65, *p* > 0.1].

If the listeners showed a build-up type of response, the first percept in each sequence should have been that of one-stream, independent of Δ*f*. In normal-hearing listeners, however, the first decision was found to be two-stream at large Δ*f* values (Deike et al., 2012). For the CI users, the probability that the first decision was a two-stream percept is shown in **Figure 3C**. As there are only two alternatives, these data also reflect the probability of the first decision being one stream, and both probabilities add up to 100%. The probability of a two-stream percept is low at small Δ*f* but rises to a mean close to 100% at large Δ*f*. As in the normal-hearing listeners, this suggests that there is no default one-stream percept in CI users. A repeated-measures analysis of variance on the factor Δ*f* (Greenhouse–Geisser corrected, 𝜀 = 0.57), showed that this effect is significant [*F*(3,21) = 5.50, *p* < 0.05].

### TIME COURSE OF PERCEPT PROBABILITIES

**FIGURE 4:**
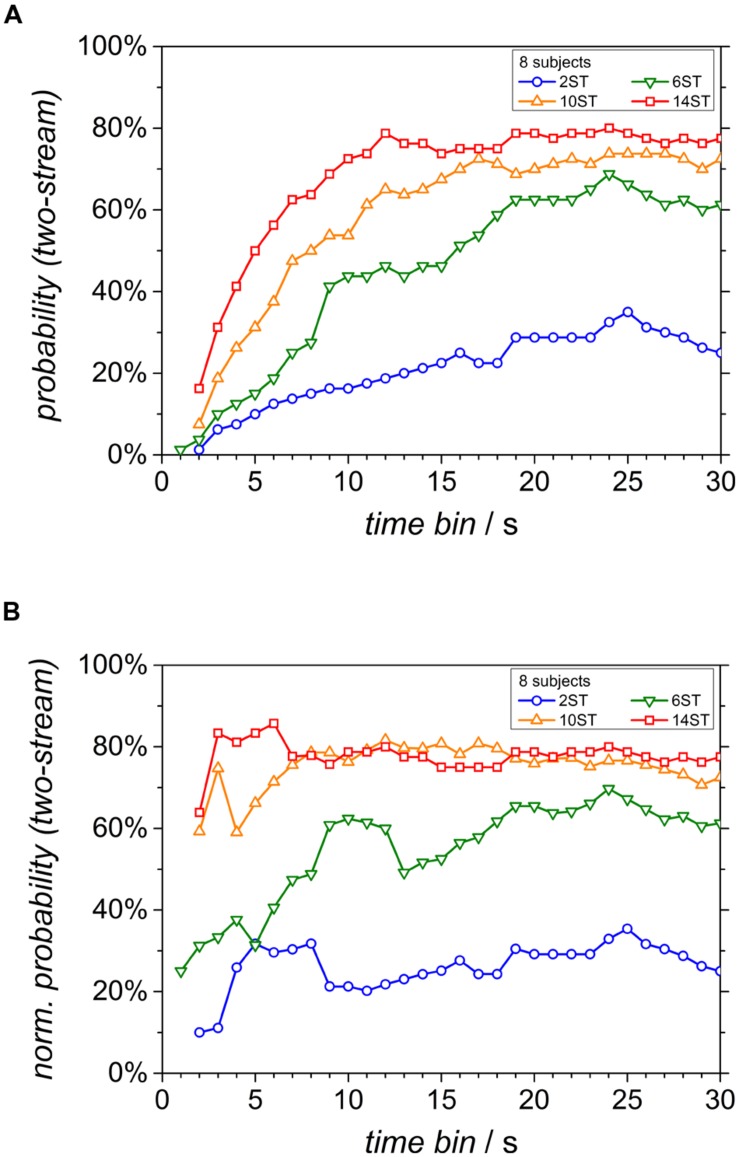
**Time courses of the current probability of the two-stream percept for the different Δ*f* conditions. (A)** Conventional display and **(B)** normalized probability, rescaled by the probability that any response has been given yet (see text).

To study the time evolution of the percept, the current probability of the two-stream percept was calculated at every instant. This is displayed in **Figure 4A**. Since only very few responses occurred within the first second for almost all frequency differences, these time courses started at 2 s for all conditions except for the Δ*f* of 6 semitones. As expected, the two-stream probability settles at higher values for larger Δ*f* (darker lines). Furthermore, all two-stream courses take several seconds to grow to the final probability value. To assess if this is indeed a build-up behavior, the two-stream data were rescaled by the probability that any response has occurred yet (i.e., the sum of the probabilities of both percepts) according to Deike et al. (2012). The course of this normalized two-stream probability reaches a maximum already after the first two seconds for the highest Δ*f* condition (**Figure 4B**). However, for lower Δ*f* values, the probability still increases slowly.

## DISCUSSION

### PERCEPTUAL SEGREGATION IN CI USERS

The present study investigated whether, with increasing frequency separation Δ*f* between the A and B harmonic complexes, CI users showed an increase of the two-stream percept that is similar to that found in normal-hearing listeners. In contrast to previous studies on stream segregation in CI users which stimulated the implant electrodes either directly (Chatterjee et al., 2006) or at the center frequencies of custom-fitted channels, the present study tested stream segregation with the same stimuli as used for normal-hearing listeners in an every-day listening situation (free-field presentation) where the listeners used their familiar CI processor settings. An additional roving by 5 semitones within the A and B sequences minimized effects due to the position of the signal frequencies with respect to the frequency setting of the CI device. The proportions of the one-stream percept and the two-stream percept resemble the results obtained with the same paradigm in normal-hearing listeners (cf. Figure 2B in Deike et al., 2012): With increasing Δ*f*, the proportion assigned to a two-stream percept increased significantly and the proportion assigned to a one-stream percept decreased. In this respect, the average results are consistent with the presence of stream segregation in CI users.

The average proportion of a two-stream percept varied from about 20% at a Δ*f* of 2 semitones to 65% at a Δ*f* of 14 semitones. The corresponding average values of normal-hearing listeners were about 5 and 80%. Thus, even at a Δ*f* of 14 semitones there remains a considerable larger proportion attributed to a one-stream percept in CI users than in normal-hearing listeners. While the chosen Δ*f* range covers both extremes of the one-stream and two-stream percepts for the normal-hearing listeners, the CI users showed on average a smaller dynamic range in their response. This is mainly due to the large inter-individual variation found in the participating CI users: Only five of the eight studied listeners responded with a monotonic change in response from one stream to two streams as the frequency separation increased. The other three listeners (CI01, CI04, and CI07) provided responses rather independent of Δ*f*, indicating that these listeners had a similar perception for all frequency differences. These individual data are not consistent with typical results of normal-hearing and hearing-impaired listeners, where the amount of segregation increased as the difference between A and B sounds increased (Rose and Moore, 2005). In the absence of stream segregation one may expect only one-stream responses. For these CI users of the present study, however, the proportions of one-stream and two-stream percepts were comparable at all Δ*f* levels. It is conceivable that, for these particular CI users, the two percepts are possible and equally probable for all conditions used in the present study, even at an average frequency separation of only 2 semitones. Alternatively, this result may be interpreted as an indication of a large uncertainty of the task. According to this interpretation, these listeners experienced a percept that is similar in all conditions but is different from segregation. Taken together, three of the eight CI listeners of this study responded in a way that is different from normal-hearing listeners, i.e., without a monotonic increase of the two-stream percept with frequency difference. Previous studies on stream segregation in CI users reported a similar ratio with three out of eight (Cooper and Roberts, 2007), or one out of five (Hong and Turner, 2006) listeners failing to show a classical stream segregation. As stated for numerous other studies on psychophysical tasks in CI users, for example music perception, it is difficult to isolate factors that might be responsible for individual performance (cf. Looi, 2008; Limb and Roy, 2014). These may be found on all stages from the coupling to the auditory nerve to the listening experience of the listeners with non-speech material.

It is noteworthy that the available CI users in the present study were all aged above 60, whereas the normal-hearing listeners of Deike et al. (2012) were young adults below 40. Thus, one may argue that the comparison was confounded by age. In agreement with this hypothesis, Rose and Moore (2005) found increased fission boundaries in some but not all bilaterally hearing-impaired listeners. Those listeners were also of higher age than the normal-hearing control listeners. However, whereas the increased fission boundaries could not be explained completely by hearing loss, there was also no evident effect of age from their data. When controlling for the hearing loss, Snyder and Alain (2007) found for the psychoacoustical performance in sequential stream segregation no effect that could be attributed to age itself. Furthermore, there is little evidence for a deterioration of a general CI performance in progressive age. Moreover, the stability of the performance over decades in several audiological tests has been shown in a large population of CI users (Lenarz et al., 2012). Therefore, the age effect has presumably only little effect in the comparison of the CI users tested here to the data of Deike et al. (2012).

The present results are in quantitative agreement with two preceding studies investigating stream segregation in CI users, showing that the occurrence of a two-stream percept requires a separation of two to three electrodes (Chatterjee et al., 2006; Cooper and Roberts, 2007). Whereas the first study did not provide information on the processor settings, the latter reported a frequency ratio of about 1.3 for a separation of two electrodes and about 1.5 for three electrodes. This corresponds to 5–7 semitones on the musical scale. The present study measured a separation of 6 semitones eliciting an ambiguous percept and 10 semitones a prevalence of segregation. Apart from the large inter-individual variability, the average data of the listeners showing stream segregation are very similar to those of the normal-hearing listeners. The fact that the most ambiguous condition with equivalent proportions of both percepts is found at 6 semitones, as in the data of normal-hearing listeners, may be surprising since the frequency difference limen in CI users is much larger than in normal-hearing listeners: In free-field presentation, on average about 3–4 semitones were found (Pretorius and Hanekom, 2008). In light of this strongly reduced frequency discrimination of CI users, one may have expected a significantly higher Δ*f* necessary to elicit stream segregation. The similarity of the data of CI users and normal-hearing listeners argues against the hypothesis that stream segregation is determined by the ability to discriminate frequencies. This is consistent with the findings of a poor correlation of the fission boundary and the frequency difference limen of hearing-impaired listeners (Rose and Moore, 2005). As the fundamental frequency increased, the fission boundary remained constant when expressed in units of equivalent rectangular bandwidth, whereas the frequency difference limen increased. Thus the ratio of fission boundary and frequency discrimination is not constant, at least not in listeners with an impaired hearing. The finding of the present study is also consistent with another study suggesting that the necessary fundamental frequency difference for stream segregation in CI users was of the order of the values found in non-musical, normal-hearing listeners (Marozeau et al., 2013).

### TIME ELAPSED BEFORE FIRST RESPONSE

The traditional hypothesis of build-up is that a two-stream percept emerges from an initial, default one-stream percept (Anstis and Saida, 1985; Carlyon et al., 2001; Boehnke and Phillips, 2005; Micheyl et al., 2005; Pressnitzer et al., 2008). Following this hypothesis, one may expect for the current experiment a shorter response time if the first percept is that of one-stream (the default) than if it is a two-stream percept. In the latter condition, the listener would have to change the impression from the default to the two-stream percept before responding. The current data do not support this hypothesis since the response time was not affected by Δ*f*. On the contrary, it was on average shorter for the 14 semitones condition than for 2 semitones.

The median response times of the CI listeners range from about 3.5–6.5 s depending on the condition. It is evident from Figure 3B in Deike et al. (2012) that except for the 6 semitones condition the normal-hearing listeners provided 50% of the responses within the first two seconds of a sequence. This corresponds roughly to the lowest quartile of the CI listeners. Therefore, the time needed for the first decision is considerably shorter for the normal-hearing listeners than for the CI users (Deike et al., 2012). It is unlikely that this is due to the sound processing in the CI device. It rather indicates perceptual differences between the two groups of listeners. The CI users seem to be generally less confident in their perceptual decision than normal-hearing listeners. This interpretation agrees well with a “context effect” reported by Marozeau et al. (2013): For the same large frequency separation between A and B tones, the difficulty rating to segregate A tones was rated as lower when the average frequency separation of the A and B tones continuously decreased from large to small than when it increased from small to large values. This effect was not observed in the normal-hearing listeners and indicates that it is more difficult for the CI users to find the appropriate cue for segregation. One should note that the time needed for the first decision was also large in normal-hearing listeners (Deike et al., 2012), considerably larger than what is commonly found in, e.g., simple pitch discrimination experiments (Ritter et al., 1972). This indicates that the task of the present study is of higher complexity than that for simple auditory discrimination.

### DO CI USERS SHOW A BUILD-UP OF SEGREGATION?

The time courses of the two types of percept based on the traditional analysis with a one-stream percept as the initial default resemble that of previous studies with normal-hearing listeners, including the data shown in Deike et al. (2012): The probability of the two-stream percept increased toward the end of the sequence (see **Figure 4A**) and is very similar to the results of normal-hearing listeners shown in Figure 3A of Deike et al. (2012). Note, however, that this slow increase cannot immediately be attributed to a build-up since it ignores the fact that any response requires some time to be given. Taking into account the probability that any response had occurred, the probability of the two-stream percept started at a high level in conditions with a high Δ*f* (**Figure 4B**), indicating that the two-stream percept prevailed immediately. This is again similar to the data of normal-hearing listeners when analyzed in this way (see in Figure 3C of Deike et al., 2012). In the normal-hearing listeners it started at about 100% two-stream percept and tended to decrease as the presentation time increased. In contrast, for the CI users, this value increased slightly within the first three seconds even for the largest Δ*f*. However, it is clearly in favor of the two-stream percept already at the moment of the first decision. One should note that the data do not necessarily reveal the perception at the very beginning of the stimulus. It could either be that the initial perception is already that of the first decision or that they do not perceive any of the alternatives (neither one nor two streams). The data of normal-hearing listeners and CI users argue against the build-up hypothesis, i.e., the initial one-stream percept which may change to a two-stream percept (Micheyl et al., 2005; Pressnitzer et al., 2008).

Consistent with the results in the normal-hearing listeners, a build-up of the two-stream percept was only observed at the 6-semitones condition, settling at a probability close to 60%, i.e., about equal prevalence of both percepts. This is consistent with the data of normal-hearing listeners. They also showed at a Δ*f* of 6 semitones a continuous increase asymptotically converging to 60%. It is also in line with the results of Denham et al. (2013). There, the initial percept was also two-stream at large frequency separations and high presentation rates. Furthermore, without normalization of the probability the time constant of build-up strongly decreased with increasing frequency separations. For CI users as well as for normal-hearing listeners, a build-up of segregation is thus restricted to a region of high ambiguity of the sequence of stimuli. The data argue against build-up as a general description for the time course of stream segregation.

In summary, the present experiment provides evidence that most of the CI users show stream segregation that is comparable with normal-hearing listeners. For large frequency differences of the A and B tones, a two-stream percept is predominant. The similarity between the results of the CI users and normal-hearing listeners indicates that the quality of stream formation is not altered when the auditory input is provided via a CI. This argues against a strong relation of stream segregation and frequency discrimination since the latter is affected by the limitations of the CI. For the experimental design of stream segregation in CI users, the present findings suggest that durations of more than 20 s are advisable to account for the prolonged time needed by the CI users to form a certain type of streaming percept.

## Conflict of Interest Statement

The study was partly supported by a research grant of MED-EL GmbH Germany, providing means for equipment and subject expenses.
